# Determination of HCV genotypes and viral loads in chronic HCV infected patients of Hazara Pakistan

**DOI:** 10.1186/1743-422X-8-466

**Published:** 2011-10-09

**Authors:** Amjad Ali, Muhammad Nisar, Habib Ahmad, Nausheen Saif, Muhammad Idrees, Mohammad A Bajwa

**Affiliations:** 1 Department of Biotechnology University of Malakand, Chakdara, Khyber Pakhtoonkhaw, Pakistan; 2 Department of Botany University of Malakand, Chakdara, Khyber Pakhtoonkhaw, Pakistan; 3 Department of Genetics, Hazara University, Mansehra-Pakistan; 4 Division of Molecular Virology, National Centre of Excellence in Molecular Biology University of Punjab Lahore, Pakistan; 5 Department of Gastroenterology, Sheikh Zayed Medical Complex, Lahore-Pakistan

## Abstract

Hepatitis C Virus (HCV) genotype and viral load are two significant predictive variables knowledge of which might persuade treatment decisions. The objective of the present study was to identify the distribution of different HCV genotypes circulating in the study area and to estimate viral load in chronically HCV infected patients. Out of total 305 HCV positive patients, 177 (58%) were males and 128 (42%) were females. Frequency breakup of the HCV positive patients was 169, 69, 38 and 29 from Abbottabad, Mansehra, Haripur and Battagram districts respectively. Out of the total 305 tested serum samples, 255 (83.06%) were successfully genotyped whereas 50 (16.4%) samples were found with unclassified genotypes. Among typable genotypes, 1a accounted for 21 (6.8%) 1b for 14 (4.6%), 2a for 4 (1.31%) 3a for 166 (54.42%) and genotype 3b for (8.19%). Twenty five (8.19%) patients were infected with mixed HCV genotypes. Viral load distribution was classified into three categories based on its viral load levels such as low (< 60, 0000 IU/mL), intermediate (60,0000-80,0000 IU/mL) and high (> 80,0000 IU/mL). The baseline HCV RNA Viral load in HCV genotype 3 infected patients was 50 (26.17%), 46 (24.08%) and 95 (49.73%) for low, intermediate and high categories respectively. For genotypes other than 3, these values for low, intermediate and high viral load categories were 50 (43.85), 35 (30.70) and 29 (25.43) respectively. Pre-treatment viral load in patients with untypable genotype was 19 (38.00%), 5 (20.00%) and 11 (44.00%) for low, intermediate and high viral load categories. Viral load distribution was also categorized sex wise; for males it was 58 (32.76%), 26 (14.68%) and 93 (52.54%) whereas for females it was 40 (31.25%), 34 (26.56%) and 54 (42.18%) for low, intermediate and high viral load respectively. In conclusion HCV genotype 3a is the most prevalent genotype circulating in Hazara Division like other parts of pakistan. Pre-treatment viral load is significantly high (p 0.014) in patients infected with HCV genotype 3 as compared to other genotypes.

## Background

Hepatitis C virus (HCV) is a principal cause of chronic liver diseases including liver fibrosis, liver cirrhosis and hepatocellular carcinoma [1,2]. Nearly 170-200 million individuals infected globally including 10-17 million persons in Pakistan [1,3-5]. HCV is an enveloped virus having positive single stranded RNA as its genome that was firstly discovered in 1989 belonging to a virus family *Flaviviridae *[3,6,7]. HCV genome is approximately 9.6 kb in length with single open reading frame and encodes a polypeptide of 3000 amino acids [8,9]. Total six major HCV genotypes and multiple subtypes have been identified from around the world [10]. Identification of HCV genotype/subtype is extremely important clinically before prescribing therapy because genotypes 1 & 4 show more resistance as compared to genotypes 2 and 3 to PEG-IFN plus ribavarin therapy, therefore different types of HCV genotypes require different duration and dose of anti viral therapy [11]. Treatment durations for genotypes 1 and 4 are 48 weeks where as for 2 and 3 is 24 weeks with PEG-IFN plus ribavarin therapy [12]. Prevalence of viral genotypes has been documented in three different patterns to date [1]. First pattern differentiate by high genetic heterogeneity involves different geographical regions of West Africa with types 1 and 2 [13], Central Africa with type 4 [14] and Asia with types 3 and 6 [15]. Second pattern entails regions with a few subtypes circulating in an intravenous drugs abusers groups, e.g., subtype 3a [16]. The last pattern involves areas where a single subtype is circulate, such as in Egypt with subtype 4a [17] and South Africa with subtype 5a [18].

Beside HCV genotypes, pre-treatment viral load has also been shown to be prognostic indicator of response to antiviral therapy [19] as increased pre-treatment viral load has been linked with low rates of response to standard interferon therapy [20-22]. Several studies have shown that patients with lower pre-treatment viral load (< 80,0000) are more likely to positively respond to currently available antiviral therapy as compared to high pre-treatment viral load (> 80,0000) [23-25]. The findings of several studies have also been indicated that a decline in HCV viral load during the first 2-12 weeks of therapy with antiviral treatment indicate to be prognostic of therapeutic efficacy [26,27]. Hence HCV genotype, baseline viral load and decrease of viral load in the stage of initial therapy play important roles in modifying and optimizing antiviral treatment [28].

In Pakistan few large scale studies are available that have been conducted in different districts, cities and towns for the identification of HCV genotypes in chronic HCV infected patients circulating in that particular areas [5,29-31]. No study is available from Pakistan that has correlated pre-treatment viral load with HCV genotypes. Therefore, this study was conducted to identify the distribution of different HCV genotypes/Subtypes and pre-treatment HCV RNA viral load in HCV infected patients belonged to different parts of Hazara Division of Pakistan.

## Methods

### Sampling

Total of 305 serum samples were collected from different chronically HCV infected patients for HCV genotyping along with specifically designed data sheets from patients visiting/attending various secondary and tertiary collection centers situated in different cities/towns of Hazara Division of Khyber Pakhtoonkhaw (KPK), Pakistan. Informed consent was taken in written form from each participated patient including, demographic characteristic, age, district, and estimated time of infection along with complete address and phone number of the patients. Written informed consent was taken from each patient.

### HCV Qualitative test

Samples were subjected firstly for the detection of HCV RNA qualitatively as previously describe by Idrees [32]. Reverse transcription PCR (RT-PCR) was done for the identification of HCV RNA. RNA was extracted from 100 μl patient's sera using Quigen RNA extraction kit according to the kit protocol. Nested PCR were performed using Taq DNA polymerase enzyme (Fermentas Technologies USA) in a volume of 20 μl reaction mix. The nested PCR products were visualized on 2% agarose gel under "UV"light using "Uvitec" gel documentation system.

### HCV Quantitative test

HCV RNA quantification was done by using SmartCycler II Real-time PCR (Cepheid, Sunnyvale, Calif. USA) with HCV RNA quantification kits (Sacace Biotechnologies, Italy). The SmartCycler II system is a PCR system by which amplification and diagnosis were accomplished at same time with TaqMan technology (Applied Biosystems, Foster City, Calif) using fluorescent probes to investigate amplification after each replicating cycle. The lower and upper detection limits of the used assay were 250 and 5.0 × 10^8 ^IU/mL, respectively. Specimens yielding values above the upper limit were diluted 100-fold, retested and the obtained values were multiplied by this dilution factor to get the actual HCV RNA concentration in international units (IU) per mL.

### HCV Genotyping

All qualitative PCR positive sera were subjected to HCV genotyping by using type-specific HCV genotyping procedure as described previously in detail [33]. Briefly, 10 μl (about 50 ng) of HCV RNA was reverse transcribed to cDNA using 100 U of M-MLV RTEs at temperature of 37°C for 50 minutes. Two μl of synthesized cDNA was used for PCR amplification of 470-bp region from HCV 5'NCR along with core region by first round PCR amplification. The amplified first round PCR product were subjected to two second rounds of nested PCR amplifications. One with mix-I primers set and the second with mix-II primers set in a reaction volume of 10 μl. Mix-I had specific genotype primers set for 1a, 1b, 1c, 3a, 3c and 4 genotypes and mix-II contain specific genotype primers set for 2a, 2c, 3b, 5a and 6a.

### Statistical Analysis

Given data was analyzed and the summary statistic was carried out by a statistical package, SPSS version 10.0. All variables results were given in the form of rates (%). Chi Square test was used for categorical variables that measured association among categorical variables. All data are presented as mean values or number of patients. P-values less than 0.05 were considered significant

## Results

### Distribution of HCV genotypes in studied patients

Table 1 shows the distribution of HCV genotypes in the studied population. Out of total 305 patients, more than 88% were male and about 42% were female. Out of the total 305 tested serum samples, type-specific PCR fragments were seen in 255 (83.06%) serum samples whereas 50 (16.4%) samples were found with untypeable genotypes as no genotype-specific band was seen in these samples. The distribution of typeable genotypes is as follows: genotype 1a accounted for 21 (6.8%), 1b for 14 (4.6%), 2a for 4 (1.31%) 3a for 166 (54.42%) and genotype 3b for 25 (8.19%) patients. Twenty five (8.19%) patients were infected by two (mixed) genotypes at a time. Predominant genotype of the study is 3a followed by 3b. The predominant genotype among males was 3 (61.58%), followed genotype 1 (11.86%), 2 (1.69%), mixed genotype (8.47%) and undetermined (16.38%). Similarly the frequent genotype among the infected female patients was 3 (64.06%) followed genotype 1 (10.39%), genotype 2 (0.78), (16.40%) and mixed genotypes (7.81%). The HCV sub-genotype pattern in males was: subtype 3a was identified in 96 (54.23%), 3b in 12 (9.37%), 1a in 11 (6.21%), 1b in 10 (5.64%) and 2a in 3 (1.69%) HCV infected patients. Among the females it was: subtype 3a in 70 (54.68%), 3b in 12 (9.37%), 1a in 10 (7.8%), 1b in 4 (3.12%) and 2a was identified in 1 (0.78%) patient.

**Table 1:** Pattern of HCV genotype among the infected patients (N = 305)

Genotype	Subtype	Male (%)	Female (%)	Total (%)
**Genotype 1**		21 (11.86%)	14 (10.93%)	35 (11.47%)

	1a	11 (6.21%)	10 (7.8%)	21 (6%)

	1b	10 (5.64%)	4 (3.12%)	14 (4%)

**Genotype 2**		3 (1.69%)	1 (0.78%)	4 (1.31%)

	2a	3 (1.69%)	1 (0.78%)	4 (1.31%)

**Genotype 3**		109 (61.58%)	82 (64.06%)	191 (62.62%)

	3a	96 (54.23%)	70 (54.68%)	166 (86.91%)

	3b	13 (7.34%)	12 (9.37%)	25 (13.08%)

**Mixed Genotypes**		15 (8.47%)	10 (7.81%)	25 (8.19%)

**Unclassified**		29 (16.38%)	21 (16.40%)	50 (16.39%)

**Total**		**177**	**128**	**305**

### Distribution of HCV genotypes in different age groups

The distribution of HCV genotypes in different age group patients is shown in table 2. Genotype 3a was the predominant genotype (45.8%) in young patients (ages between 31-40 years). Frequent prevalence was found in age group of 31-40

**Table 2:** Pattern of HCV genotypes/subtypes in different age groups (N = 305)

	Age groups (in years)					
**Genotype/Subtype**	**10-20**	**21-30**	**31-40**	**41-50**	**51-60**	**> 60**

1a	0	8 (38.08%)	3 (14.3%)	8 (38.08%)	2 (9.0%)	0

1b	0	3 (21.42%)	7 (50%)	2 (14.5%)	2 (14.5%)	0

2a	1 (25%)	1 (25%)	1 (25%)	0	1(25%)	0

3a	2 (1.2%)	33 (19.9%)	76 (45.8%)	32 (19.3%)	15 (9.03%)	8 (4.81%)

3b	2 (8%)	8 (32%)	7 (28%)	6(24%)	1 (4%)	1 (4%)

Mixed genotypes	4 (16%)	4 (16%)	8 (32%)	5 (20%)	3 (12%)	1(4%)

Unclassified	7 (28%)	7 (28%)	21 (24%)	8 (16%)	6 (12%)	1(2%)

Total	16 (5.25%)	64 (20.32%)	123(40.32%)	61(20%)	30(9.83%)	11 (3.61%)

### Distribution of HCV genotypes in different parts of Hazara

Table 3 demonstrates the prevalence of HCV genotypes that were recorded from HCV patients belonged to different parts of Hazara Division. Among the typeble genotypes 169 belonged to Abbottabad. Among the genotypes samples from Abbottabad 12 (7.10%) were 1a, 10 (5.10%) were 1b, 100 (59.17%) were 3a, 12 (7.10%) were untypable and 26 (15.38%) were with mixed genotypes. Genotypes distribution among the patients of Mansehra was 69. Among these, 4 (5.79%) were 1a, 2 (2.8%) were 1b, 40 (57.97%) were 3a, 10 (3.55%) were 3b and 8 (11.59%) were with mixed genotypes. Five (7.24%) were untypeable. Out of 38 samples that were genotyped in HCV positive sera from Haripur, 3 (7.89%) were 1a, 18 (47.36%) were 3a, 4 (10.52%) were 3b, 8 (21.05%) were with mixed genotypes and 3 (7.89%) were undetermined. Prevalence of HCV genotypes among Battagram positive patients were 29 in which 2 (6.89%) were 1a, 1 (3.44%) was 2a, 8 (27.58%) were 3a, 5 (17.24%) were 3b, 8 (27.58%) were with double infection and 5 (17.24%) were unclassified HCV.

**Table 3:** Prevalence of HCV genotypes in different geographic regions of Hazara (N = 305)

Genotype	Sub-type	Isolated from Abbottabad	Isolated from Mansehra	Isolated from Haripur	Isolated from Battagram	P Value
1	1a	12(7.10%)	4(5.79%)	3(7.89%)	2(6.89%)	NS

	1b	10(5.90%)	2(2.8%)	2(5.26%)	0(0.00%)	NS

2	2a	3(1.77%)	0(0.00%)	0(0.00%)	1(3.44%)	NS

3	3a	100(59.17%)	40(57.97%)	18(47.36%)	8(27.58%)	NS

	3b	6(3.55%)	10(14.49%)	4(10.52%)	5(17.24%)	< 0.05

Mixed	_	26(15.38%)	8(11.59%)	8(21.05%)	8(27.58%)	0.05

Unclassified	_	12(7.10%)	5(7.24%)	3(7.89%)	5(17.24%)	> 0.05

**Total**		**169**	**69**	**38**	**29**	

### HCV RNA viral loads in male and female patients with different genotypes

Viral load was classified into three categories based on its level such as low (< 60, 0000 IU/ml), intermediate (60,0000-80,00000 IU/ml) and high (> 80,00000 IU/ml) viral load. Base line HCV Viral load for each genotype in both male and female patients are shown in table 4. Pre-treatment viral load was found high significantly (p 0.014) in patients infected with HCV genotype 3 as compared to other genotypes. No significant difference was observed in male and female HCV infected patients (p 0.343).

**Table 4:** HCV RNA viral load categories and their distribution in sex and genotype in studied population

Genotype/Subtype	Viral load			P value
	< 60,0000	60,0000-80,0000	> 80,0000	

Genotype 3	50(26.17%)	61(32.00%)	80(41.88%)	
Other genotypes	50(43.85%)	35(30.70%)	29(25.43%)	0.014

Male	58(32.76%)	57(32.24%)	62(35.02%)	
Female	40(31.25%)	51(39.84%)	37(28.90%)	0.343

**Abbottabad**				

Genotype 3	25(23.58%)	41(35.68%)	40(37.73%)	
Other genotypes	26(41.26%)	19(30.15%)	18(28.57%)	0.053

**Mansehra**				

Genotype 3	20(40%)	17(34%)	13(26%)	
Other	7(36.87%)	8(42.10%)	4(21.6%)	0.809

**Haripur**				

Genotype 3	2(9.09%)	11(53.02%)	8(36.36%)	
Other	6(31.57%)	5(31.25%)	4(25%)	0.094

**Battagram**				

Genotype 3	3(23.07)	6(46.15%)	4(30.76%)	
Other	8(50%)	5(31.25%)	3(18.25%)	0.32

## Discussion

Hazara Division is a historical and beautiful locality of the province Khyber Pakhtoonkhaw (KPK) province of Pakistan. This part of the world has been mapped such that on the North and East lies the Northern Areas and Azad Kashmir, to the South are the Islamabad Capital Territory and the province of Punjab, while the rest of KPK lies to its West. The river forms majority of the western border of the Division by running in a North-south fashion. Hazara Division is one of the most diverse regions based on the ethnic groups it is comprised of, such as Awan, Dhund Abbassi, Gabari, Ghakkar, Gujjar, Jadoon, Karlal, Karlugh, Kohistanis, Maddakhel, Mishawani, Paracha, Shilmani, Syed, Swati, Tahirkheli, Tanoli, Tareen, Turks, Kashmiri, Dilazak and the Isazai Pashtoon respectively [34].

In this study we have studied the distribution of HCV genotypes and associated these genotypes with gender. The results of this study clearly showed that there is no variation among the HCV genotypes and gender as different HCV genotypes were distributed with same ratio between males and females. In agreement with this observation of our study, no significant difference was seen by Idrees and Riazuddin [5] in gender and variation among genotypes in this part of the world. On the other hand in contrast to our observation, in Libya HCV genotypes were not distributed with same pattern between males and females as seen in Pakistan. In Libya, the prevalence of HCV genotype 1 was found to be significantly associated with males while genotype 4 was seen frequently in females [35]. Our results indicated that high prevalence rate 40.32% of HCV infection was found between age group of 30-40. These findings are in agreement with the findings of Ahmad *et al*, [29] that highest rate of prevalence was observed in age group of ≤ 40 years and similar findings were also observed by Inamullah *et al*, [30]. But our findings contradict from that of Muhammad *et al*, [36] that high HCV prevalence rate in Pakistan was found in old age group people. So these results suggest that early diagnosis of HCV might be due to the awareness of public about HCV infection in this part of the world. Currently a number of studies have been carried out to find the frequencies of various HCV genotypes in different geographical regions of Pakistan and the most prevalent genotype was 3a [5,28,31,37]. Data analysis of the present study showed that genotype 3a (54.42%) has high prevalence rate followed by 3b and 1a in chronic HCV infected patients. These results confirmed the findings of other studies reported from Pakistan that the most prevalent genotype is 3a followed by genotype 3b and 1a, respectively [27,6,5,33,38]. Identified HCV genotypes in our study showed no regional difference and was distributed with same fashion predominant genotype was 3a followed by 3b as has also been reported by Ali et al., [27] from various regions of Pakistan where the authors concluded that there is no regional difference in HCV genotype distribution. Our pattern of HCV genotypes distribution is similar to that reported from neighboring countries like in Iran and India where predominant genotype is 3 [39,40], and is different from South Asian countries such as Japan and Thailand where genotype 1 is the common HCV genotype [41,42].

In the present study 8.19% of the studied patients were found infected by two different genotypes at the same time, majority of these were thalassaemic patients who had received unsafe blood in past. Our results are supported by other studies that show that mixed genotypes are more frequent where blood transfusion is common especially in thalassaemic patients [27]. Franciscus [21] states that mixed genotypes in a single patient may affect the antiviral therapy response and disease succession. We were unable to identify HCV genotypes 4, 5 and 6 in the current study. This observation confirmed the previously reported arguments that these three genotypes are not existing in this region or these are partially absent from Pakistan [27,28].

It has already been reported that patients with higher viral load show lower response rates to standard antiviral therapy as compared to patients having lower viral load [21]. Our findings carry some important implication for the therapeutic hindrance that genotype 3 is the most common genotype in Pakistan [5] and also in our studied patients. Untimely detection and treatment are significant to achieve a high level of sustained virological response (SVR) [43]. Early time detection involves the identification of low HCV RNA level [11]. As determined by Von et al., [21] and Dalgard et al, [22], that shorter therapy schedules told that genotype 3 HCV infected patients with low baseline viral load (HCV-RNA, < 600, 000-800, 000 IU/mL) had more likely to attained a sustained virological response (SVR) as compared to those with a high viral load (HCV-RNA > 60000-800000 IU/mL). We found only 50 (26.17%) patients infected by genotype 3 in our studied patients that had intermediate viral load (< 80,0000) after HCV RNA quantification. As per the research findings Von *et al*. [21], patients having genotype 3 and with high viral load (> 80,0000 IU/mL) should be treated for 24 weeks where as patients with low (< 80,0000 IU/mL) RNA viral load might be treated for 16 weeks for those patients whose HCV RNA PCR is undetectable at week 4 of treatment. Our findings along with those of Von et al. [21] further recommend that extensive information about HCV genotype and basal RNA viral load is necessary when planning therapy strategies against HCV at national level. These results also help to modify antiviral therapy individually for infected HCV patients with genotype 3 that will reduce economic burden, side effects of antiviral therapy, and also may promote optimize response rates. Finally the results of this study show that more than one genotype of HCV circulates in Hazara Division of KPK, Pakistan. Pre dominant genotype was 3a followed by 3b and 1a.

## Conclusions

In the present study, we conclude that HCV genotype 3a is the most prevalent genotype circulating in this region of the world. Regional difference do exists in HCV genotypes. Majority of the infected patients are young ages between 31-40 years. Baseline viral load is significantly high in patients infected by HCV genotype 3 (subtypes a & b) as compared to other genotypes such as 1 (subtypes a, b, c), 2 (subtypes a, b) and untypeable genotypes.

## Competing interests

The authors declare that they have no competing interests.

## Authors' contributions

AA and MN conceived the study. AA and N collected the samples and performed the molecular genotyping analysis. AA searched the literature and drafted the manuscript. MI critically reviewed the manuscript. All the authors read and approved the final manuscript.
